# Mosquito- and biting-midge-borne arboviruses in Western Yunnan’s border region, China

**DOI:** 10.1186/s13071-026-07400-6

**Published:** 2026-05-27

**Authors:** Yiling Yang, Yuwen He, Yantao Zhu, Jing Wu, Keping Zhao, Zhao Li, Zhengxing Yang, Jianzhong Yin, Jinglin Wang

**Affiliations:** 1 Yunnan Key Laboratory of Cross-Border Infectious Disease Prevention and New Drug Development and Yunnan Provincial Key Laboratory of Public Health and Biosafety, School of Public Health, Kunming Medical University, Kunming, China; 2 Department of Medical Genetics, Institute of Medical Biology, Chinese Academy of Medical Sciences and Peking Union Medical College, Kunming, Yunnan China; 3 Yunnan Tropical and Subtropical Animal Viral Disease Laboratory, Yunnan Animal Science and Veterinary Institute, Kunming, China; 4 Kunming Medical University Haiyuan College, Kunming, Yunnan China; 5 Mangshi City Animal Disease Prevention and Control Center, Mangshi, China; 6 Jiangcheng County Animal Disease Prevention and Control Center, Jiangcheng, China

**Keywords:** Mosquitoes, Biting midges, Arboviruses, Isolation, Identification

## Abstract

**Background:**

Arboviruses that infect humans and other mammals pose significant public health challenges worldwide, particularly in developing countries. The border region of Yunnan Province, a key entry point for imported arboviruses into China, is characterized by a subtropical climate and a rich diversity of mosquito and biting midge species, making it a high-risk area for arbovirus transmission. While some studies have focused on mosquito-borne viruses in this region, research on midge-borne viruses, particularly those with zoonotic relevance, remains limited. This study aimed to systematically investigate the diversity of arboviruses in both vector groups and to address critical gaps in the assessment of spillover risk.

**Methods:**

In July 2018, mosquitoes and biting midges were collected from Mangshi, Yingjiang, Lushui, and Tengchong in the border region of western Yunnan Province, China. Following morphological classification, specimens were pooled for virus isolation using BHK-21 and C6/36 cells. Viral isolates were identified by reverse transcription polymerase chain reaction (RT-PCR) using virus-specific primers, and sequence analyses were performed using Clustal X, DNASTAR, and MEGA-X.

**Results:**

A total of 8581 mosquitoes (8 species in 4 genera) and 18,298 biting midges (19 species) were collected. *Culex tritaeniorhynchus* dominated the mosquito populations in Mangshi (92.11%) and Yingjiang (97.23%), whereas *Anopheles sinensis* was predominant in Lushui (44.01%) and Tengchong (59.23%). Biting midge composition varied among regions, with *Culicoides tainanus* predominating in Mangshi (59.79%) and *C. arakawai* in Lushui (48.11%). In total, 189 viral strains representing 10 virus species in 8 families were isolated. Tibet orbivirus (TIBOV) was the most widely distributed virus (83 strains), occurring in all four regions, with biting midges accounting for most isolates (50/83 strains). Biting midges also contributed substantially to arbovirus diversity, yielding 13 Yunnan orbivirus (YUOV) strains, 4 Banna virus (BAV) strains, and the only Akabane virus (AKAV) isolate. In contrast, mosquitoes mainly harbored insect-specific viruses (ISVs), including 53 Manglie virus (MAV) strains. Novel viruses such as Culex-originated Tymoviridae-like virus (CuTLV) and Armigeres iflavirus (ArIFV) were identified in both vectors. These findings highlight biting midges as important yet understudied vectors, harboring distinct zoonotic and vector-associated viruses in this border region.

**Conclusions:**

This study demonstrates the circulation of diverse arboviruses in both mosquitoes and biting midges in the border region of western Yunnan Province. Notably, biting midges harbored multiple arboviruses, including several viruses with zoonotic potential or known pathogenicity, such as Tibet orbivirus (TIBOV), Yunnan orbivirus (YUOV), Banna virus (BAV), and Akabane virus (AKAV). These findings emphasize the important role of biting midges, alongside mosquitoes, in maintaining and potentially spreading these viruses in the local arboviral ecosystem. The study underscores the importance of including midge-borne viruses in future surveillance efforts, as they contribute substantially to the diversity and epidemiology of arboviral transmission in the region, with potential implications for public health.

**Graphical Abstract:**

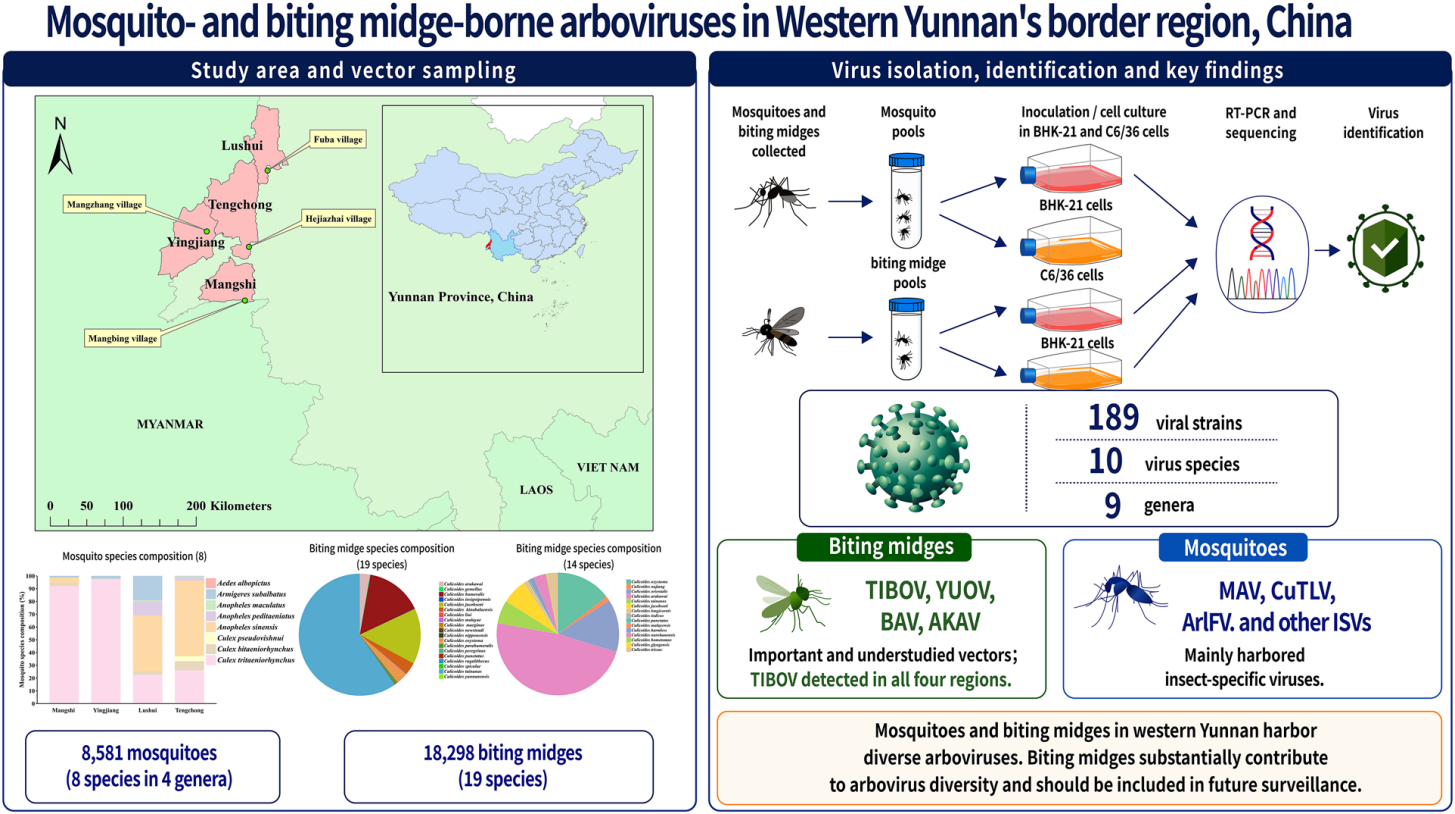

**Supplementary Information:**

The online version contains supplementary material available at 10.1186/s13071-026-07400-6.

## Background

Arthropod-borne viruses (arboviruses) represent a growing global health threat, particularly in tropical and subtropical regions, where climate change, human migration, and increased economic exchanges accelerate their emergence and spread [1, 2]. More than 600 arboviruses have been identified, many of which cause significant diseases in humans and animals, including dengue virus (DENV) and Japanese encephalitis virus (JEV) [3, 4]. DENV, first isolated in 1943, now circulates in more than 100 countries worldwide [5]. JEV, the leading cause of viral encephalitis in Asia, infects an estimated 69,000 individuals annually, with a 20–30% mortality rate among severe cases [6, 7]. While mosquitoes (*Culicidae*) are well documented as the primary vectors of many arboviruses [8], biting midges (Ceratopogonidae) are increasingly recognized for their role in zoonotic disease transmission. More than 50 viruses transmitted by biting midges, including bluetongue virus (BTV), African horse sickness virus (AHSV), and Akabane virus (AKAV), significantly impact livestock and agriculture [9, 10]. Furthermore, insect-specific viruses (ISVs), which do not infect vertebrates, are prevalent in vector populations and may influence the transmission dynamics of pathogenic arboviruses by modulating vector competence [11, 12]. Similarly, midge-specific viruses (MSVs) have also been identified in *Culicoides* populations through metagenomic and transcriptomic studies. Recent investigations in Yunnan Province revealed a diverse range of insect-specific viruses (ISVs) in biting midges, including members of the families *Nodaviridae, Partitiviridae, Totiviridae*, and *Iflaviridae* [13]. Unlike arboviruses that replicate in both insect vectors and vertebrate hosts, these midge-specific viruses are restricted to their insect hosts and are believed to be maintained primarily through vertical transmission. Although their biological significance remains largely unexplored, emerging evidence suggests that insect-specific viruses may modulate vector competence by influencing the replication and transmission of pathogenic arboviruses through phenomena such as superinfection exclusion. The growing recognition of midge-specific viruses highlights the need for comprehensive virome surveillance in *Culicoides* populations, particularly in arbovirus-endemic regions such as Yunnan Province.

Yunnan Province, located in southwestern China, is a high-risk hotspot for arboviral transmission due to its subtropical monsoon climate, rich biodiversity, and its 4061-km border with Vietnam, Laos, and Myanmar [14]. These geographic and climatic factors facilitate the proliferation of vector species and the cross-border exchange of pathogens [1]. Over the past three decades, Yunnan has reported the emergence of multiple novel arboviruses, often linked to trade and population movement with Southeast Asia [15]. Despite the region’s significance in the context of arboviral diseases, research has predominantly focused on mosquitoes [16–19], with biting midges, particularly those transmitting zoonotic viruses that affect livestock, remaining underexplored.

In this study, we conducted a comprehensive survey of mosquitoes and midges from four western border regions of Yunnan—Mangshi, Yingjiang, Tengchong, and Lushui—in 2018. Through a combination of morphological identification, viral isolation, and phylogenetic analysis, our study provides a detailed understanding of the diversity of arboviruses in midge populations, evaluates their potential role in zoonotic spillover, and offers valuable data to inform public health strategies aimed at controlling arboviral transmission in the region. This research highlights the importance of addressing both mosquito and midge-borne pathogens in the development of more effective vector control programs and the prevention of arboviral outbreaks.

## Methods

### Sample collection

**Fig. 1:**
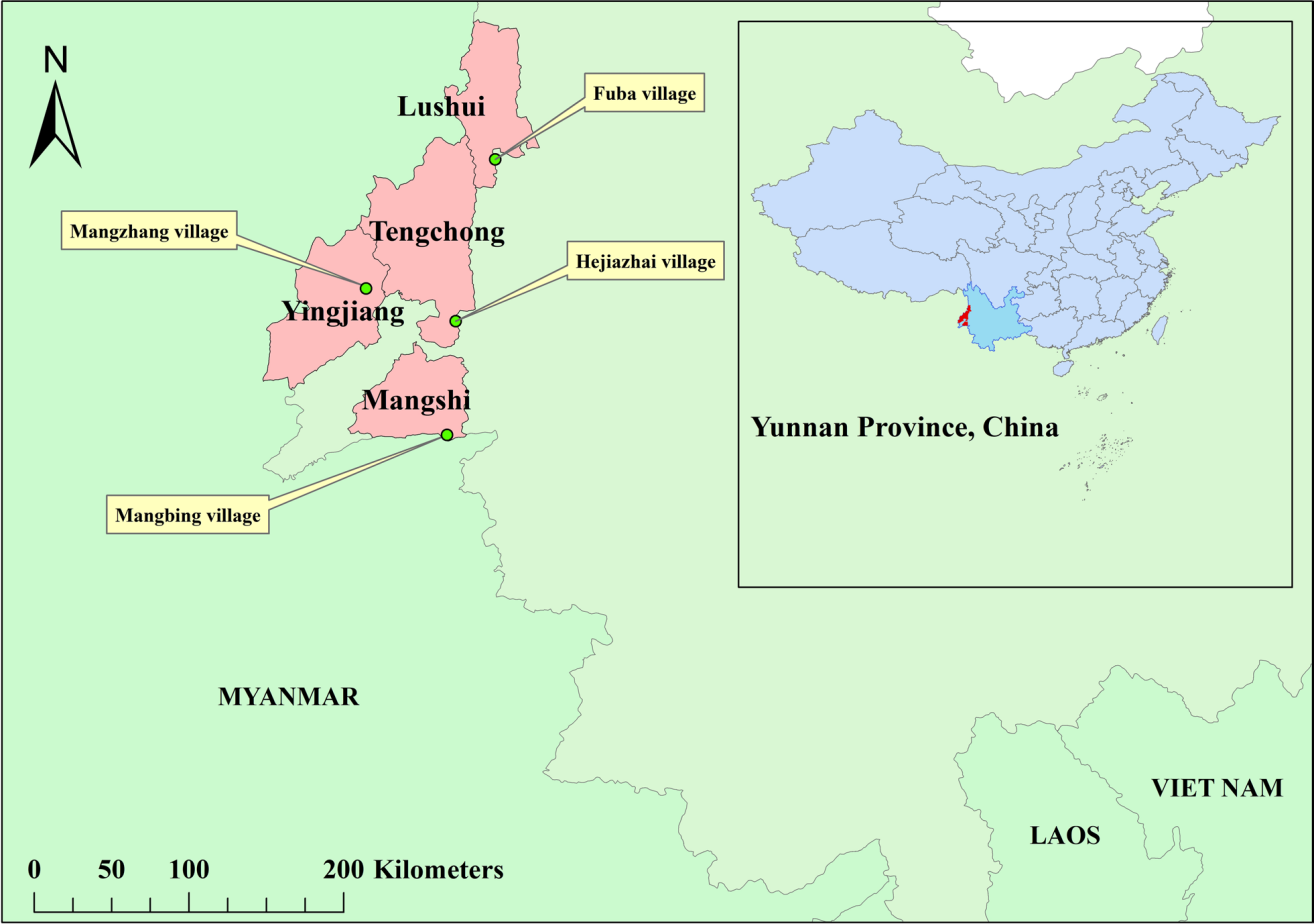
Geographical locations of the sampling sites in the western border region of Yunnan Province, China

From 23–26 July 2018, mosquitoes and biting midges were collected from multiple sites in the western border region of Yunnan Province, China. The sampling sites included a dairy farm and a cattle cooperative in Mangbing Village, Mangshi (24°6′N, 98°35′E); a cattle farm and a pig farm in Mangzhang Village, Yingjiang County (24°41′N, 97°58′E); fish ponds, pigpens, and cattle sheds in Hejiazai Village, Tengchong City (24°43′N, 98°27′E); and pigpens, chicken coops, and cattle pens in Fuba Village, Lushui City (25°42′N, 98°52′E) (Fig. 1).

Mosquitoes and biting midges were collected using mosquito traps (Kongfu Xiaoshuai, 12 V, 300 mA; Wuhan, Hubei, China). The traps were operated daily from 6:00 PM to 7:00 AM the following morning. At each sampling site, one trap was deployed per night and positioned 1.5–1.8 m above the ground in sheltered locations near animal enclosures. After collection, the insects were immobilized at −20 °C for 20 min. Specimens were then sorted and morphologically identified under a dissecting microscope. Mosquitoes were identified to species level, whereas biting midges were initially sorted as *Culicoides* spp. for routine pooling and virus isolation. Representative specimens were subsequently identified to species level for species-composition analysis. Sex was not recorded during pooling. Finally, the samples were pooled into separate tubes according to species (for mosquitoes) or genus (for biting midges), with approximately 50 mosquitoes or 100 biting midges per tube, and stored in liquid nitrogen for subsequent virus isolation.

### Virus isolation

Prior to inoculation, 24-well plates containing confluent monolayers of BHK-21 and C6/36 cells, together with the homogenization medium, were transferred into a biosafety level 2 laboratory. Inside a biosafety cabinet, the wells were sequentially numbered and the original culture medium was removed. Subsequently, 150 µL of the homogenization medium was added to each well of both cell lines, while 150 µL of MEM was added to the negative-control wells. The plates were gently agitated to ensure thorough mixing and then placed in an incubator for viral adsorption. After 2 h of incubation, the inoculum was discarded and 1 mL of maintenance medium was added to each well. The plates were returned to the incubator for further cultivation. This procedure constituted the first passage of virus isolation. Each passage was observed daily for at least 7 days, and the time of appearance and characteristics of any cytopathic effect (CPE) were recorded. At the end of the 7-day observation period, the cell culture plates were subjected to three freeze–thaw cycles at −80 °C. The lysates were then used to inoculate the next passage. Both cell lines were blindly passaged for a total of five passages. From the third passage onward, the culture supernatant from C6/36 cells was also inoculated onto BHK-21 monolayers. This was designated as the first passage of the C6/36-to-BHK-21 transfer, and this procedure was carried out for at least three passages. When CPE reached approximately 75%, the supernatant was collected, transferred to a sterile centrifuge tube, and stored at −80 °C for future use. Virus isolates were identified using virus-specific reverse transcription polymerase chain reaction (RT-PCR). Viral RNA was extracted from 200 µL of viral supernatant using TRIzol reagent (TaKaRa), yielding an eluted volume of 35 µL. Complementary DNA (cDNA) was synthesized using the M-MLV (RNase H-) Reverse Transcriptase kit (TaKaRa), following the manufacturer’s instructions. Primers targeting the following viruses or genera were selected on the basis of published literature: Densovirus (DNV), Banna virus (BAV), Tibet orbivirus (TIBOV), Yunnan orbivirus (YUOV), *Alphavirus*, and *Flavivirus*. Additionally, primers specific to Negevirus and Nam Dinh virus (NDiV) were designed using reference genome sequences (Table S1).

The pool positivity rate was calculated as (number of positive pools/total number of pools) × 100%. To enable comparison between vector groups with different pool sizes, the minimum infection rate (MIR) was also calculated as (number of positive pools/total number of specimens tested) × 1000 assuming one infected specimen per positive pool. This provides a conservative estimate of the lower bound of infection prevalence.

The PCR amplification mixture (25 µL total volume) contained 0.5 µL each of forward and reverse primers, 2.0 µL of 2.5 mM dNTPs, 2.5 µL of 10 × Ex Taq Buffer, 0.15 µL of Ex Taq polymerase (TaKaRa), 2.5 µL of cDNA template, and 16.85 µL of RNase-free ddH₂O. The thermal cycling conditions were as follows: initial denaturation at 94 °C for 5 min; 30 cycles of denaturation at 94 °C for 30 s, annealing at 50–60 °C for 30 s, extension at 72 °C for 30–75 s (Table S1); and a final extension at 72 °C for 10 min. PCR products were analyzed by electrophoresis on a 1% agarose gel. Positive bands were purified and sent to Shanghai Sangon Biotech Co., Ltd. for sequencing.

### Virus identification

Virus isolates were identified using virus-specific reverse transcription polymerase chain reaction (RT-PCR). Viral RNA was extracted from 200 µL of viral supernatant using TRIzol reagent (TaKaRa), yielding an eluted volume of 35 µL. Complementary DNA (cDNA) was synthesized using the M-MLV (RNase H–) Reverse Transcriptase kit (TaKaRa), following the manufacturer’s instructions. Primers targeting the following viruses or genera were designed on the basis of published literature: Densovirus (DNV), BAV, TIBOV, YUOV, *Alphavirus*, and *Flavivirus*. Additionally, primers specific to Negevirus and NDiV were designed using reference genome sequences (Table S1).

The PCR amplification mixture (25 µL total volume) contained 0.5 µL each of forward and reverse primers, 2.0 µL of 2.5 mM dNTPs, 2.5 µL of 10 × Ex Taq Buffer, 0.15 µL of Ex Taq polymerase (TaKaRa), 2.5 µL of cDNA template, and 16.85 µL of RNase-free ddH₂O. The thermal cycling conditions were as follows: initial denaturation at 94 °C for 5 min; 30 cycles of denaturation at 94 °C for 30 s, annealing at 50–60 °C for 30 s, extension at 72 °C for 40–75 s; and a final extension at 72 °C for 10 min. PCR products were analyzed by electrophoresis on a 1% agarose gel. Positive bands were purified and sent to Shanghai Sangon Biotech Co., Ltd. for sequencing.

### Sequence alignment and analysis

Initial sequence assembly was performed using SeqMan in the DNASTAR Lasergene software package (version 7.1.0; DNASTAR Inc., Madison, WI, USA). The assembled sequences were compared with the GenBank database using the National Center for Biotechnology Information Basic Local Alignment Search Tool (NCBI BLAST) tool to identify viral species. Relevant reference sequences were subsequently downloaded. Nucleotide and amino acid sequence alignments, together with homology analyses, were conducted using Clustal X (version 2.1) and the MegAlign module of the DNASTAR software suite. Maximum-likelihood (ML) phylogenetic trees were constructed using MEGA-X software with 1000 bootstrap replicates to assess branch support.

## Results

### Mosquito species and population composition

**Fig. 2:**
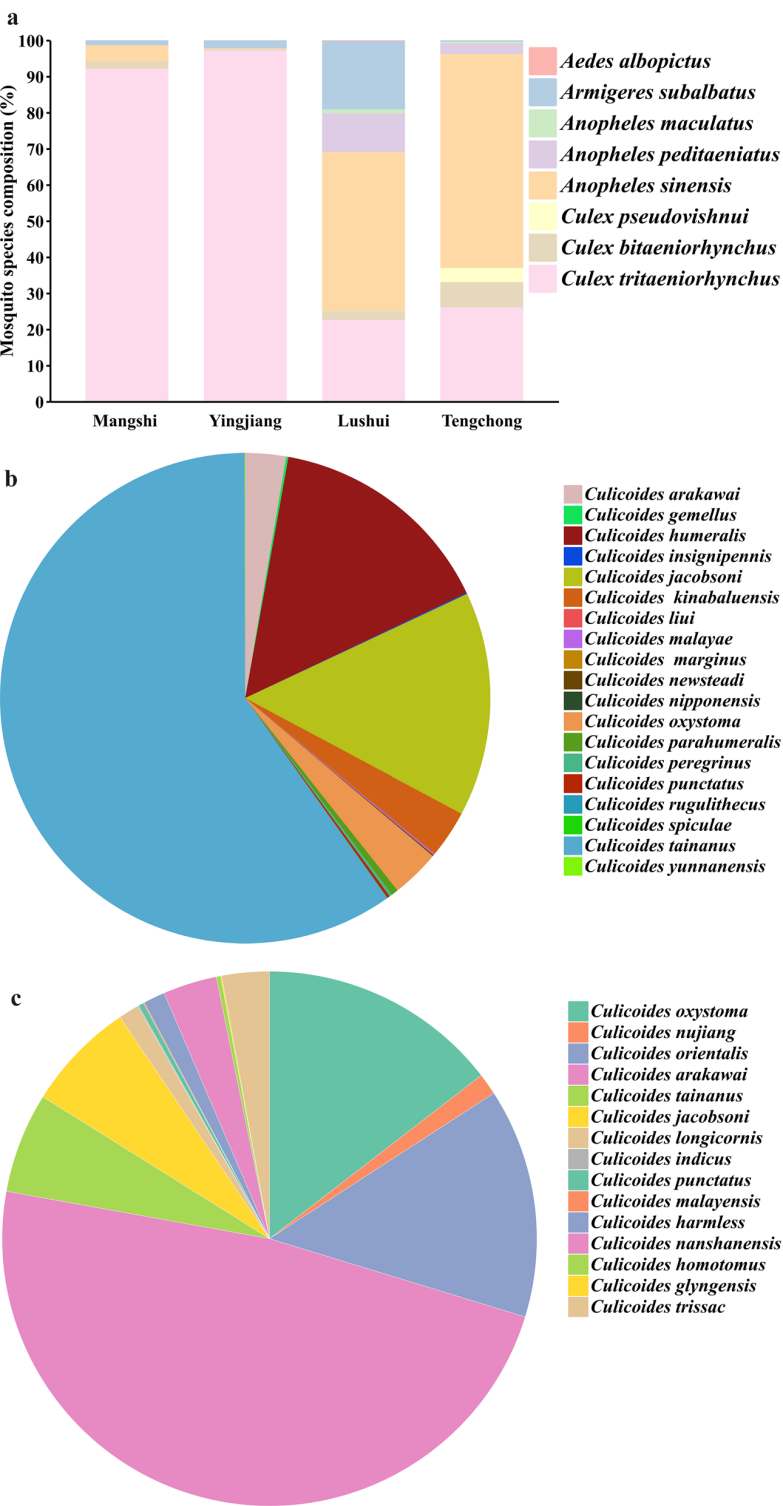
**a**. Mosquito species composition at different sampling sites in the western border region of Yunnan Province, China. **b**. Culicoides species composition in Mangshi. **c**. *Culicoides* species composition in Lushui

Biting midges were collected from all four sampling sites: Mangshi, Yingjiang, Lushui, and Tengchong. A total of 18,298 biting midges were collected across all sites. All biting midges collected were morphologically identified as belonging to the genus *Culicoides*; no other biting midge genera were detected in this survey. In July 2018, a total of 8581 mosquitoes representing 8 mosquito species in 4 genera were collected from Mangshi, Yingjiang, Lushui, and Tengchong in border regions of western Yunnan Province: *Culex*, *Anopheles*, *Armigeres*, and *Aedes*. *Culex tritaeniorhynchus* was the dominant mosquito species in both Mangshi and Yingjiang, comprising 92.11% and 97.23% of the total mosquito population at those sites, respectively. In contrast, *Anopheles sinensis* was the most prevalent species in Lushui and Tengchong, accounting for 44.01 and 59.23% of the total mosquito populations in those regions, respectively (Fig. 2A).

### Biting midge species and population composition

In July 2018, a total of 7396 biting midges representing 19 species were collected in Mangshi. The five most prevalent species were *C. tainanus* (59.79%, 4422/7396), *Culicoides humeralis* (15.10%, 1117/7396), *Culicoides jacobsoni* (14.79%, 1094/7396), *C. oxystoma* (3.20%, 237/7396), and *Culicoides kinabaluensis* (3.11%, 230/7396). The remaining *Culicoides* species each contributed less than 5% to the total population (Fig. 2B).

In Lushui, 4602 biting midges of 15 species were collected. The top five species were *Culicoides arakawai* (48.11%, 2214/4602), *C. oxystoma* (14.54%, 669/4602), *Culicoides orientalis* (13.89%, 639/4602), *C. jacobsoni* (6.65%, 306/4602), and *C. tainanus* (6.06%, 279/4602). The remaining *Culicoides* species each had a composition ratio of less than 5% (Fig. 2C).

### Virus isolation and identification

For virus isolation, mosquitoes were first grouped by species, collection site, and collection date, with each species-specific group subsequently divided into pools of approximately 50 individuals, yielding a total of 170 pools from 8581 mosquitoes. Biting midges were initially sorted as *Culicoides* spp. for pooling and were grouped by collection event (same trap and night), with each pool containing approximately 100 individuals, resulting in 203 pools from 18,298 biting midges. From these pools, a total of 189 virus strains representing 10 virus species from 8 families and 9 genera were detected, including 83 Tibet orbivirus (TIBOV), 33 Yunnan orbivirus (YUOV), 6 Banna virus (BAV), 5 Nam Dinh virus (NDiV), 1 Akabane virus (AKAV), 53 Manglie virus (MAV), 2 Yunnan Culex flavivirus (YCxFV), 4 Culex pipiens pallens densovirus (CppDNV), 1 Culex-originated Tymoviridae-like virus (CuTLV), and 1 Armigeres iflavirus (ArIFV) (Table 1).

**Table 1:** Virus isolation from mosquitoes and biting midges in western Yunnan

Collection site (county/district)	Sample type	Sample size	Isolation batch	Total	Pool positivity rate	MIR (‰)
Mangshi	Mosquitoes	4408	88	42	47.73%	9.53
	*Culicoides*	7396	85	47	55.29%	6.35
Yingjiang	Mosquitoes	941	16	0	0.00%	0.00
	*Culicoides*	1100	11	2	18.18%	1.82
Lushui	Mosquitoes	534	11	4	36.36%	7.49
	*Culicoides*	4602	55	11	20.00%	2.39
Tengchong	Mosquitoes	2698	55	3	5.45%	1.11
	*Culicoides*	5200	52	3	5.77%	0.85
Total	Mosquitoes	8581	170	49	28.82%	5.71
	*Culicoides*	18,298	203	63	31.03%	3.44

Isolation batch refers to the number of pools processed for virus isolation from each sample type and location. Total indicates the number of pools that yielded viral isolates. Pool positivity rate was calculated as (number of positive pools/total number of pools) × 100%. Minimum infection rate (MIR) was calculated as (number of positive pools/total number of specimens tested) × 1000, assuming one infected specimen per positive pool. This provides a conservative estimate of infection prevalence and allows for comparison between vector groups with different pool sizes (50 mosquitoes versus 100 biting midges per pool)

A total of 33 strains of TIBOV, 20 strains of YUOV, 2 strains of BAV, 1 strain of NDiV, 29 strains of MAV, 2 strains of CppDNV, 2 strains of YCxFV, and 2 strains of CuTLV were isolated from mosquitoes (including *Cx. tritaeniorhynchus*, *An. sinensis*, and *Cx. pseudovishnui*). Additionally, 50 strains of TIBOV, 13 strains of YUOV, 4 strains of BAV, 1 strain of AKAV, 4 strains of NDiV, 24 strains of MAV, 2 strains of CppDNV, and 1 strain of ArIFV were isolated from *Culicoides* (including *C. arakawai*, *C. oxystoma*, *C. tainanus*, *C. humeralis*, *C. jacobsoni*, and undetermined *Culicoides* spp.) (Table 2).

**Table 2:** Identification results of arboviruses in border areas of western Yunnan

		TIBOV	YUOV	BAV	AKAV	NDiV	MAV	CppDNV	YCxFV	CuTLV	ArIFV
Mosquitoes	*Cx. tritaeniorhynchus*	30	20	2	0	0	29	2	2	1	0
	*An. sinensis*	1	0	0	0	1	0	0	0	0	0
	*Cx. pseudovishnui*	2	0	0	0	0	0	0	0	0	0
*Culicoides*	*C. arakawai*	4	0	4	1	1	1	0	0	0	1
	C*. oxystoma*	3	0	0	0	3	0	0	0	0	0
	*C. tainanus*	21	13	0	0	0	15	2	0	0	0
	*C. humeralis*	9	0	0	0	0	8	0	0	0	0
	*C. jacobsoni*	8	0	0	0	0	0	0	0	0	0
	Undetermined *Culicoides* spp.	5	0	0	0	0	0	0	0	0	0
	Total	83	33	6	1	5	53	4	2	1	1

### Identification of the *Orbivirus*

#### Identification of the Tibet orbivirus (TIBOV)

**Fig. 3:**
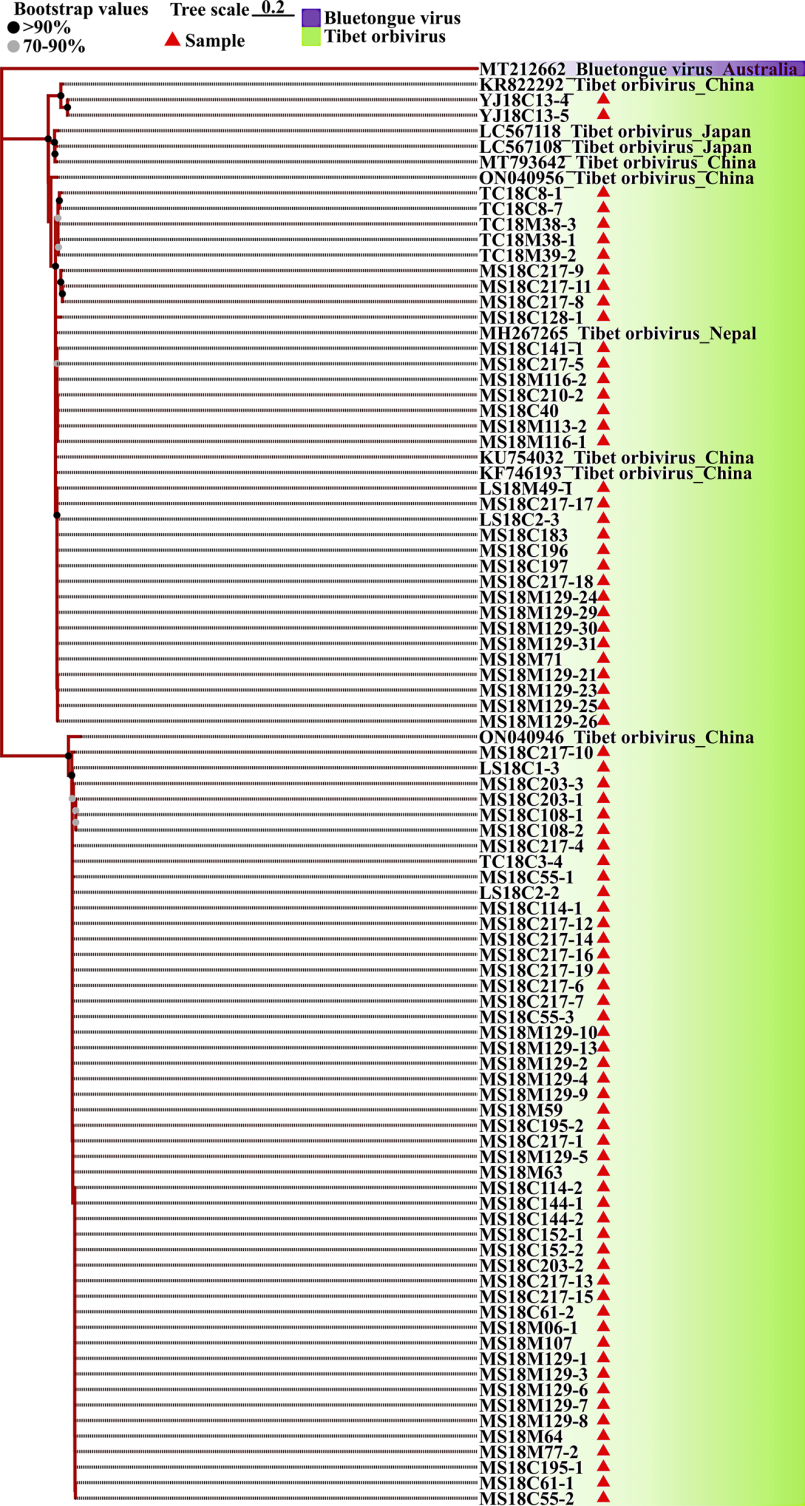

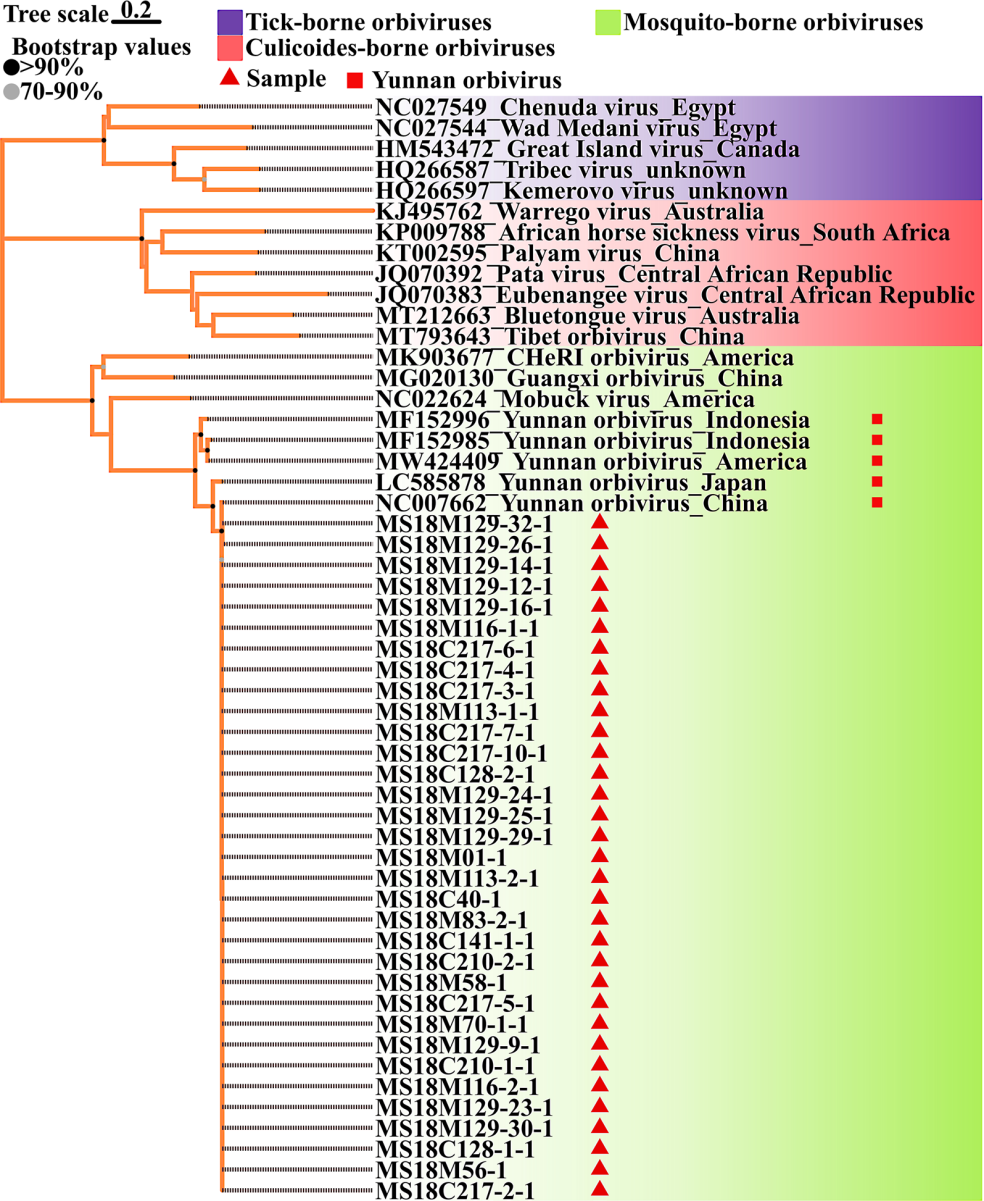
**a** Maximum-likelihood phylogenetic tree based on partial nucleotide sequences of the VP7 gene of Tibet orbivirus (TIBOV) and related orbiviruses (480 bp). Red triangles indicate the newly isolated TIBOV strains identified in this study. The best-fit maximum-likelihood evolutionary model was the Kimura 2-parameter model with a proportion of invariant sites (K2 + I). Bootstrap values were calculated from 1000 replicates. **b**. Maximum-likelihood phylogenetic tree based on partial nucleotide sequences of the VP7 gene of Yunnan orbivirus (YUOV) (440 bp). Red triangles indicate the newly isolated YUOV strains identified in this study, and red squares indicate the reference YUOV strains. The best-fit maximum-likelihood evolutionary model was the general time reversible model with gamma-distributed rate heterogeneity and a proportion of invariant sites (GTR + G + I). Bootstrap values were calculated from 1000 replicates

The identification of TIBOV was carried out using VP7-specific primers, which successfully amplified 83 strains. The VP7 gene sequences obtained, consisting of 480 nucleotides, exhibited nucleotide homology ranging from 70.0% to 99.8%. A representative strain, selected from these isolates, was used alongside the corresponding sequences of TIBOV strains downloaded from the GenBank database to construct a phylogenetic tree (Fig. 3A).

The best-fit DNA model was identified as the Kimura 2-parameter model with a proportion of invariant sites (K2 + I). Phylogenetic analysis revealed that the newly isolated strains from this study clustered within the same evolutionary branch as the known TIBOV strains, indicating that these isolates are indeed TIBOV. Further analysis showed that the TIBOV strains formed two major phylogenetic branches. The strains LS18C1-3, MS18C114-1, MS18C203-1, MS18C217-16, and MS18M129-1 clustered with the V290/YNSZ strain from Shizong County, Yunnan Province, suggesting a close evolutionary relationship. The remaining strains grouped into a separate evolutionary branch, indicating the potential presence of multiple genotypes or subtypes of TIBOV in the western border regions of Yunnan Province.

### Identification of the Yunnan orbivirus (YUOV)

For YUOV identification, VP7-specific primers were used to amplify positive isolates from 33 strains of *Cx. tritaeniorhynchus* and *C. tainanus* collected in Mangshi. The sequences of the VP7 gene, consisting of 440 nucleotides, exhibited nucleotide homology ranging from 91.8% to 100%. Phylogenetic analysis showed that the representative strains isolated in this study clustered within the same evolutionary branch as five other YUOV strains from Yunnan, Japan, and Indonesia. The new strains were most closely related to the YOV-77–2 strain from Yunnan Province, with nucleotide homology ranging from 97.1% to 99.1% (Fig. 3B).

### Identification of the Banna virus (BAV)

**Fig. 4:**
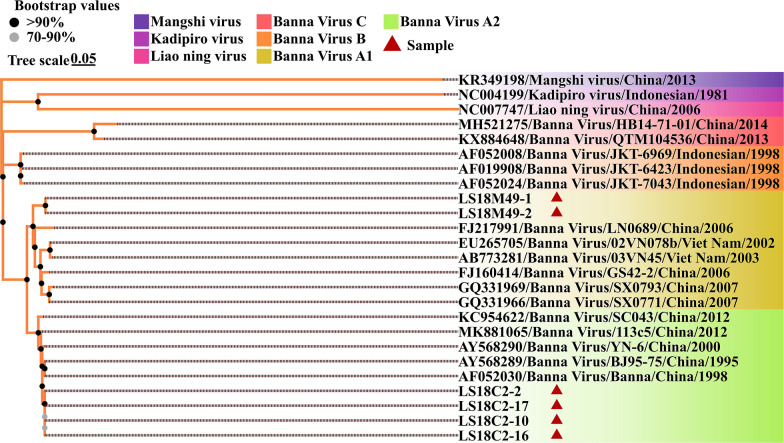
Maximum-likelihood phylogenetic tree based on nucleotide sequences of the VP12 gene of Banna virus (BAV). Red triangles indicate the newly isolated BAV strains identified in this study. The best-fit maximum-likelihood evolutionary model was the Tamura 3-parameter model with gamma-distributed rate heterogeneity and a proportion of invariant sites (T92 + G + I). Bootstrap values were calculated from 1000 replicates

For BAV identification, VP12-specific primers were used to amplify and obtain the nucleotide sequences of the VP12 gene (820 nt in length) from six positive isolates. These isolates were derived from two pools of *Cx. tritaeniorhynchus* and four pools of *C. arakawai* collected in Lushui. The sequences exhibited nucleotide homology ranging from 91.9% to 100%. Phylogenetic analysis revealed that all newly isolated strains clustered within the previously defined A-type BAV group, sharing nucleotide identities greater than 91.4% with other A-type strains. Within this cluster, two distinct sublineages were observed: one (A1-type) included strains LS18M49-1 and LS18M49-2, and the other (A2-type) comprised strains LS18C2-2, LS18C2-10, LS18C2-16, and LS18C2-17 (Fig. 4).

### Identification of the Akabane virus (AKAV)

**Fig. 5:**
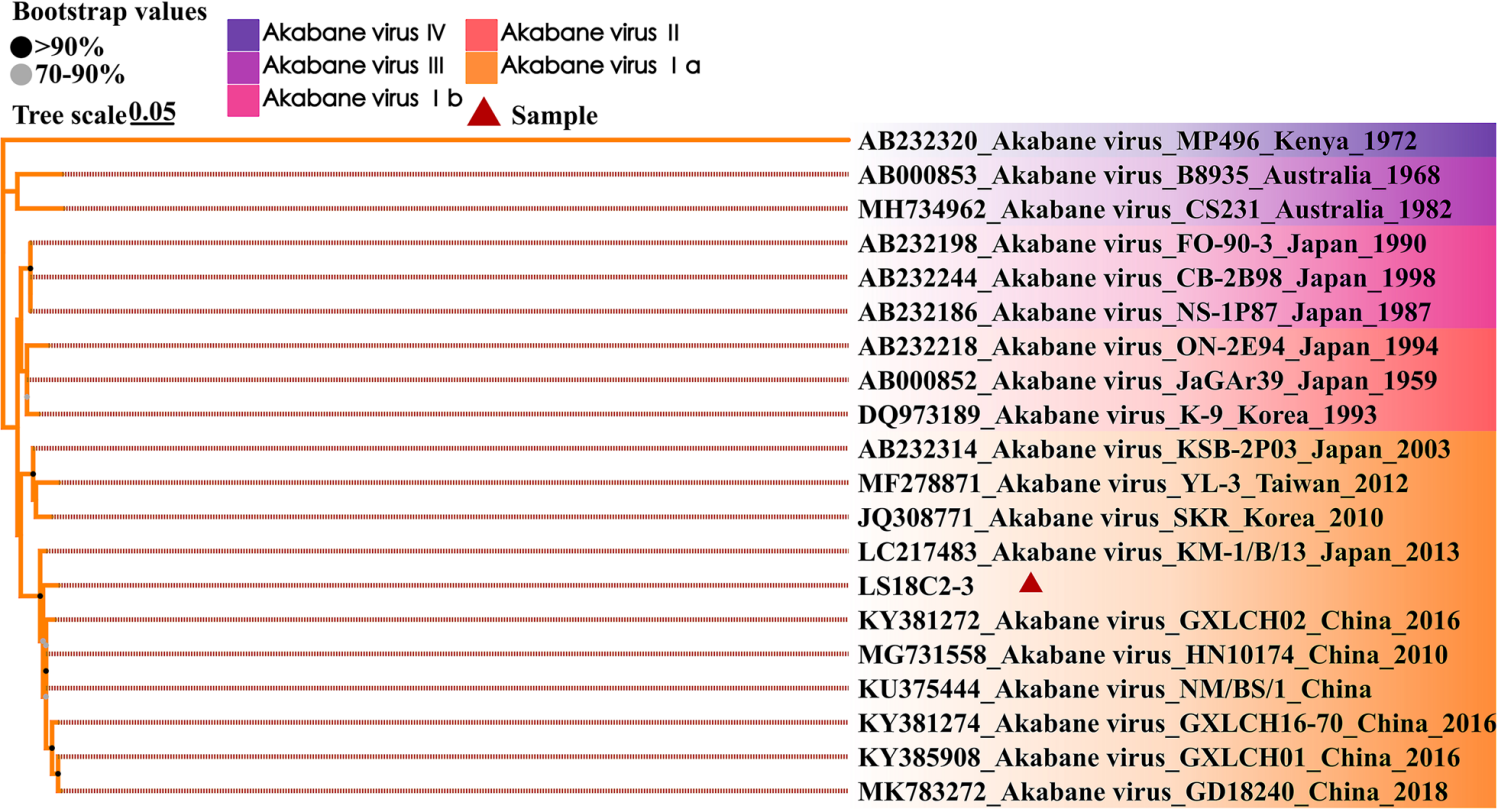
Maximum-likelihood phylogenetic tree based on nucleotide sequences of the S gene of Akabane virus (AKAV). The red triangle indicates the newly isolated AKAV strain identified in this study. The best-fit maximum-likelihood evolutionary model was the Kimura 2-parameter model with a proportion of invariant sites (K2 + I). Bootstrap values were calculated from 1000 replicates

For AKAV identification, S gene-specific primers were used to amplify a positive isolate from a pool of *C. arakawai* collected in Lushui. A 287-nucleotide sequence of the S gene was obtained and designated as strain LS18C2-3. The sequence showed 98.9% nucleotide homology to the AKAV-HN10174 strain previously isolated in Hunan Province. Phylogenetic analysis indicated that LS18C2-3 clustered within genotype Ia along with ten other AKAV strains, with nucleotide homology among them ranging from 92.7% to 97.9% (Fig. 5).

### Identification of the insect-specific viruses (ISVs)

#### Identification of the Nam Dinh virus (NDiV)

**Fig. 6:**
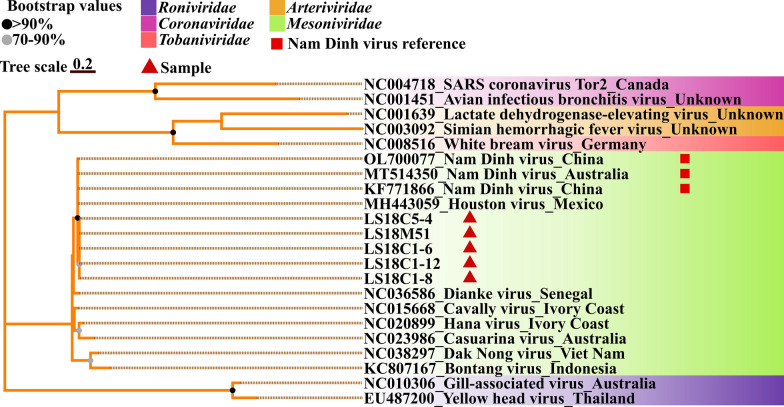
Maximum-likelihood phylogenetic tree based on partial amino acid sequences of the RNA-dependent RNA polymerase (RdRp) of Nam Dinh virus (NDiV) and related nidoviruses. Red triangles indicate the newly isolated NDiV strains identified in this study, and red squares indicate the reference NDiV strains. The best-fit maximum-likelihood evolutionary model was the Tamura 3-parameter model with gamma-distributed rate heterogeneity (T92 + G). Bootstrap values were calculated from 1000 replicates

For NDiV identification, five strains were isolated from three pools of *C. oxystoma*, one pool of *Cx. pseudovishnui* Colless, and one pool of *C. arakawai* collected in Lushui. These strains showed greater than 96.4% nucleotide homology to the reference strain NDiV-NJ8-09 (GenBank: KF771866) from Yunnan Province. Phylogenetic analysis revealed that the newly isolated strains were most closely related to three previously reported NDiV strains from Yunnan, with amino acid homology exceeding 95.8%, and clustered within the same evolutionary branch as NDiV, Houston virus (HOUV), and Dianke virus (DKV) (Fig. 6).

### Identification of the Manglie virus (MAV)

**Fig. 7:**
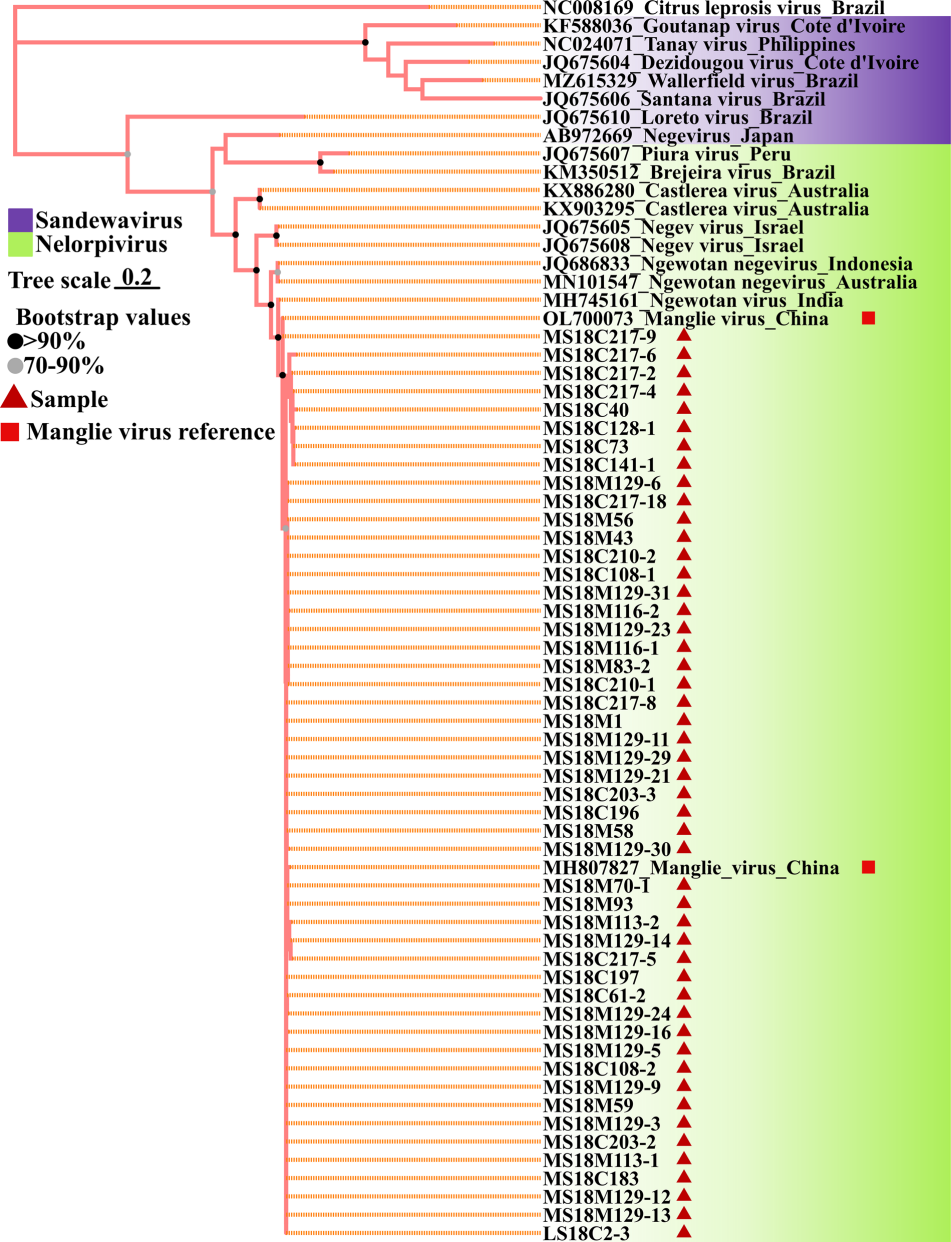
Maximum-likelihood phylogenetic tree based on partial amino acid sequences of the RNA-dependent RNA polymerase (RdRp) of Manglie virus (MAV) and related negeviruses. Red triangles indicate the newly isolated MAV strains identified in this study, and red squares indicate the reference MAV strains. The best-fit maximum-likelihood evolutionary model was the Le and Gascuel model with gamma-distributed rate heterogeneity (LG + G). Bootstrap values were calculated from 1000 replicates

For MAV identification, 53 strains were isolated from 29 pools of *Cx. tritaeniorhynchus* and 24 pools of *Culicoides* collected in Mangshi and Lushui. The nucleotide homology among these strains ranged from 95.9% to 99.3%. Phylogenetic analysis showed that all newly isolated strains clustered within the Nelorpivirus branch and were most closely related to two previously reported MAV strains from Yunnan Province (Fig. 7).

### Identification of the Culex pipiens pallens densovirus (CppDNV)

**Fig. 8:**
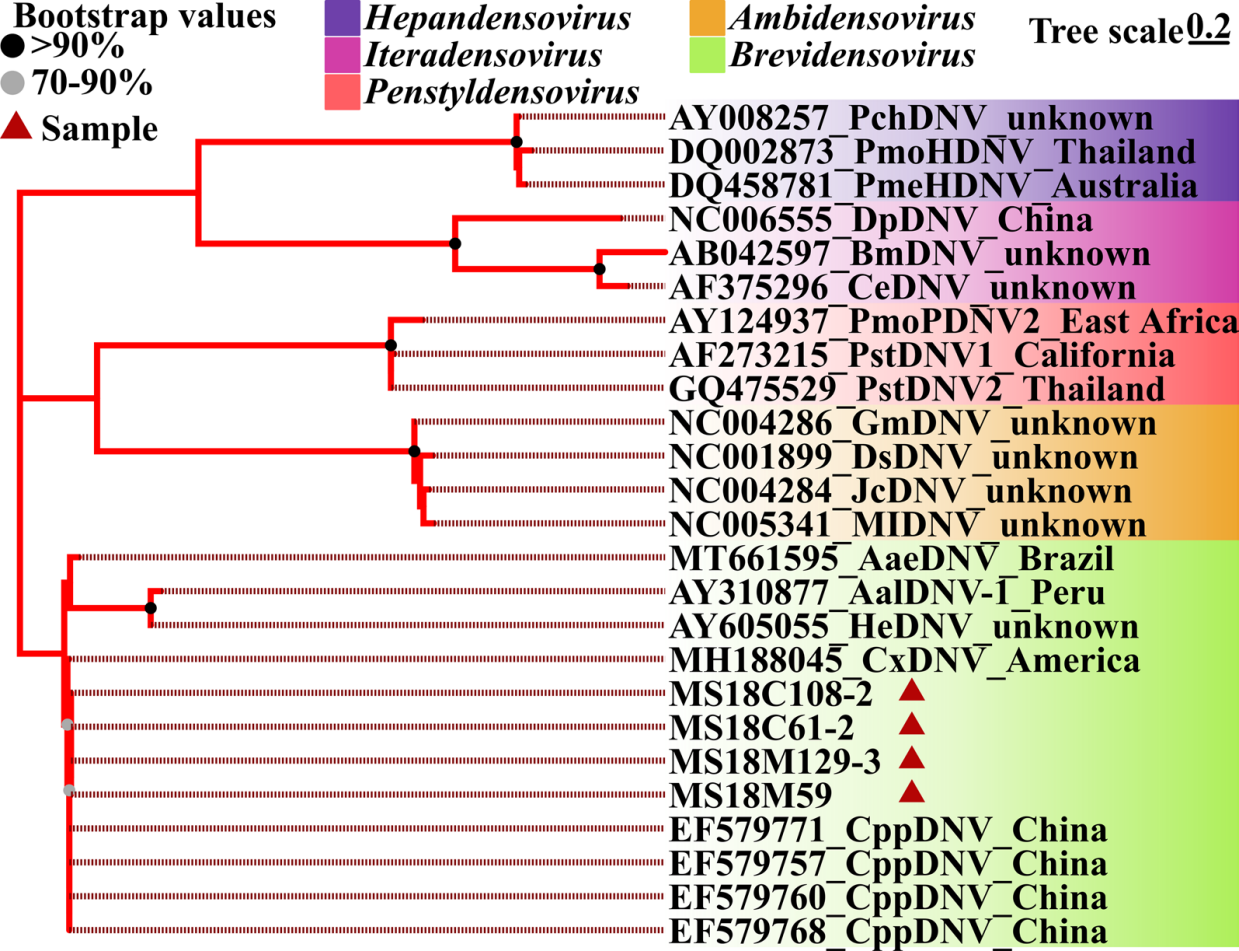
Maximum-likelihood phylogenetic tree based on partial amino acid sequences of the NS2 gene of Culex pipiens pallens densovirus (CppDNV). Red triangles indicate the newly isolated CppDNV strains identified in this study. The best-fit maximum-likelihood evolutionary model was the Le and Gascuel model (LG). Bootstrap values were calculated from 1000 replicates. PchDNV Penaeus chinensis hepandensovirus, PmoHDNV Penaeus monodon hepandensovirus, PmeHDNV Penaeus merguiensis hepandensovirus, DpDNV Dendrolimus punctatus densovirus, BmDNV Bombyx mori densovirus 5, CeDNV Casphalia extranea densovirus, PmoPDNV2 Penaeus monodon penstyldensovirus 2, PstDNV1 Penaeus stylirostris penstyldensovirus 1, PstDNV2 Penaeus stylirostris penstyldensovirus 2, GmDNV Galleria mellonella densovirus, DsDNV Diatraea saccharalis densovirus, JcDNV Junonia coenia densovirus, MIDNV Mythimna loreyi densovirus, AaeDNV, Aedes aegypti densovirus, AalDNV-1 Aedes albopictus densovirus 1, *HeDNV* Haemagogus equinus densovirus, CxDNV Culex pipiens densovirus, CppDNV Culex pipiens pallens densovirus

For *Densovirus* identification, four strains were obtained from two pools of *Cx. tritaeniorhynchus* and two pools of *C. tainanus* collected in Mangshi. These strains shared nucleotide homology ranging from 99.5% to 100%, and showed the closest relationship to the reference strains CppDNV-YN05217, CppDNV-GZWN1, and CppDNV-XJ057, with amino acid homology exceeding 97.2% (Fig. 8).

### Identification of Yunnan Culex flavivirus (YCxFV)

**Fig. 9:**
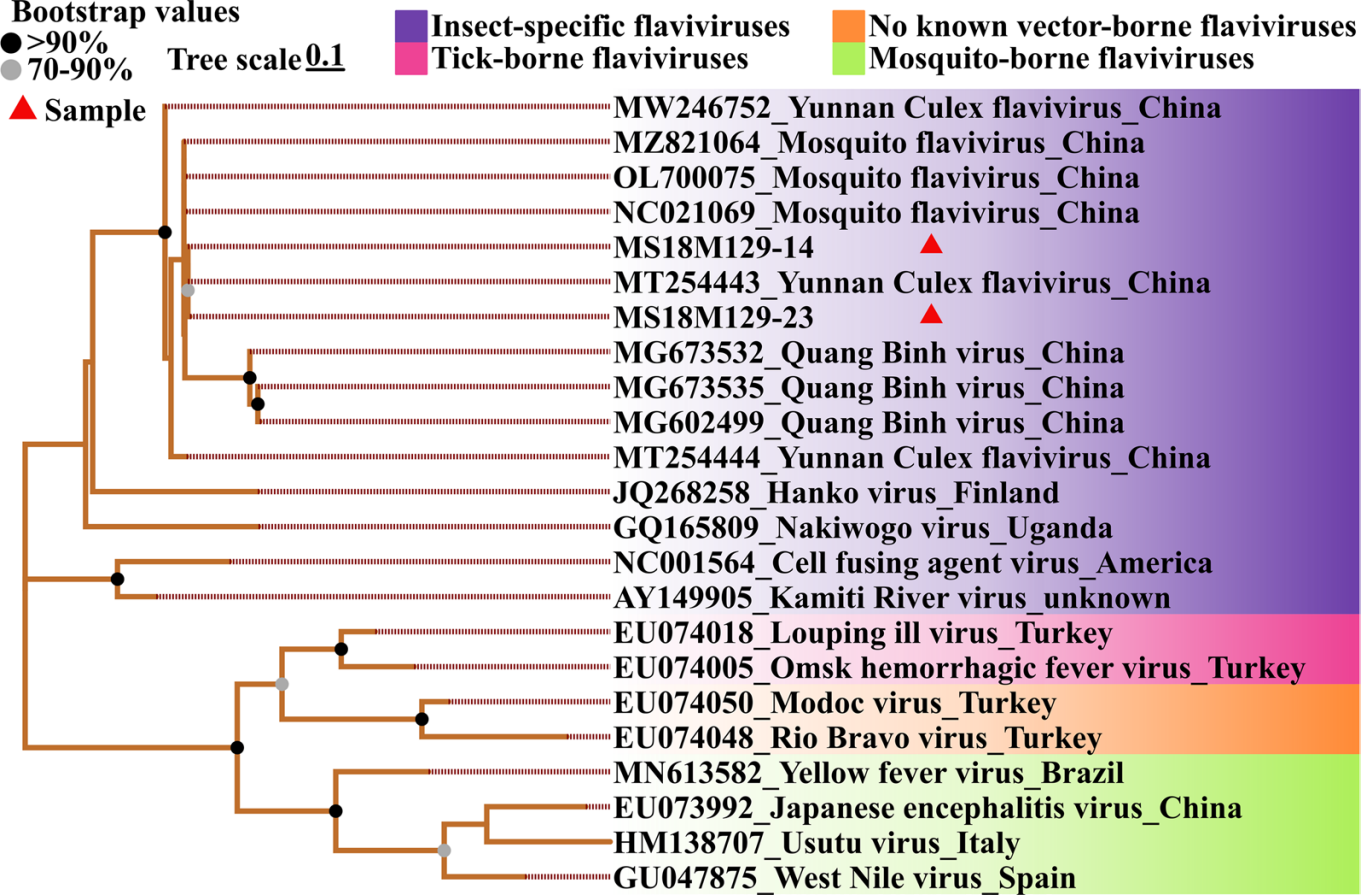
Maximum-likelihood phylogenetic tree based on partial nucleotide sequences of the NS5 gene of Yunnan Culex flavivirus (YCxFV) and related flaviviruses (272 bp). Red triangles indicate the newly isolated YCxFV strains identified in this study. The best-fit maximum-likelihood evolutionary model was the Kimura 2-parameter model with gamma-distributed rate heterogeneity (K2 + G). Bootstrap values were calculated from 1000 replicates

For YCxFV identification, two strains were isolated from two pools of *Cx. tritaeniorhynchus* collected in Mangshi, sharing 98.3% nucleotide homology between them. Phylogenetic analysis indicated that the newly isolated strains MS18M129-14 and MS18M129-23 clustered within the evolutionary branch of unclassified flaviviruses. They were most closely related to the strain MT254443 Yunnan *Culex* flavivirus, which was isolated in Yunnan Province in 2018, with nucleotide homologies of 99.5% and 98.5%, respectively (Fig. 9).

### Identification of the Culex-originated Tymoviridae-like virus (CuTLV)

**Fig. 10:**
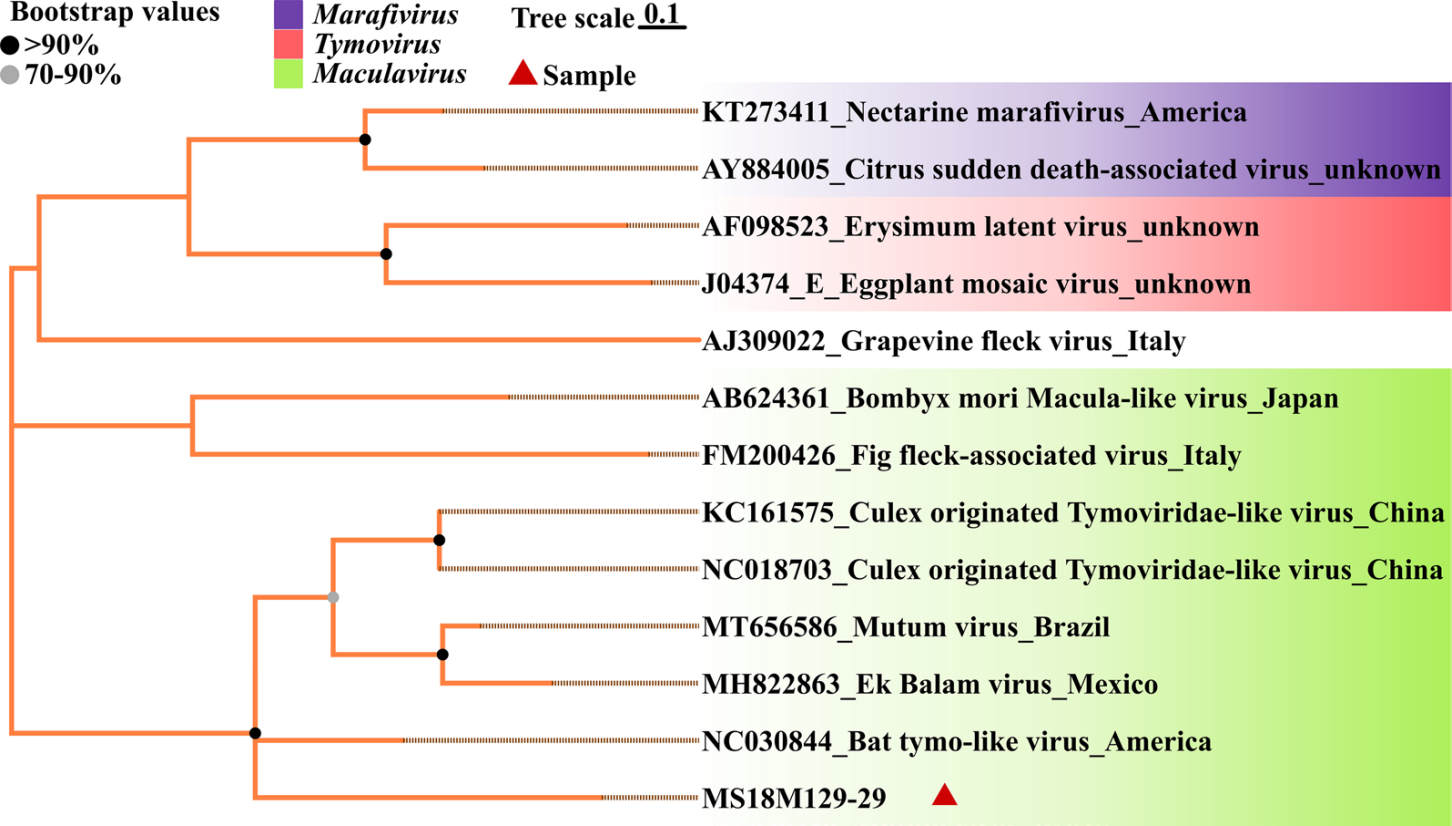
Maximum-likelihood phylogenetic tree based on partial amino acid sequences of the RNA-dependent RNA polymerase (RdRp) of Culex-originated Tymoviridae-like virus (CuTLV) and related viruses in the family *Tymoviridae*. The red triangle indicates the newly isolated CuTLV-related strain identified in this study. The best-fit maximum-likelihood evolutionary model was the Le and Gascuel model with gamma-distributed rate heterogeneity and a proportion of invariant sites (LG + G + I). Bootstrap values were calculated from 1000 replicates

For CuTLV identification, strain MS18M129-29 was isolated from a pool of *Cx. tritaeniorhynchus* and amplified using KHV S10F/R primers. BLASTx analysis revealed that it shared 61.6% amino acid identity with Culex-originated Tymoviridae-like virus (CuTLV; GenBank: NC018703), which was isolated in Xinjiang in 2005. A phylogenetic tree was constructed using the partial amino acid sequences of the RdRp gene from the MS18M129-29 strain and 12 other reference strains of the family *Tymoviridae*. The results showed that all 13 viruses formed 3 distinct clusters, with the MS18M129-29 strain clustering within the *Maculavirus* group and showing the closest relationship to CuTLV (Fig. 10).

### Identification of the Armigeres iflavirus (ArIFV)

**Fig. 11:**
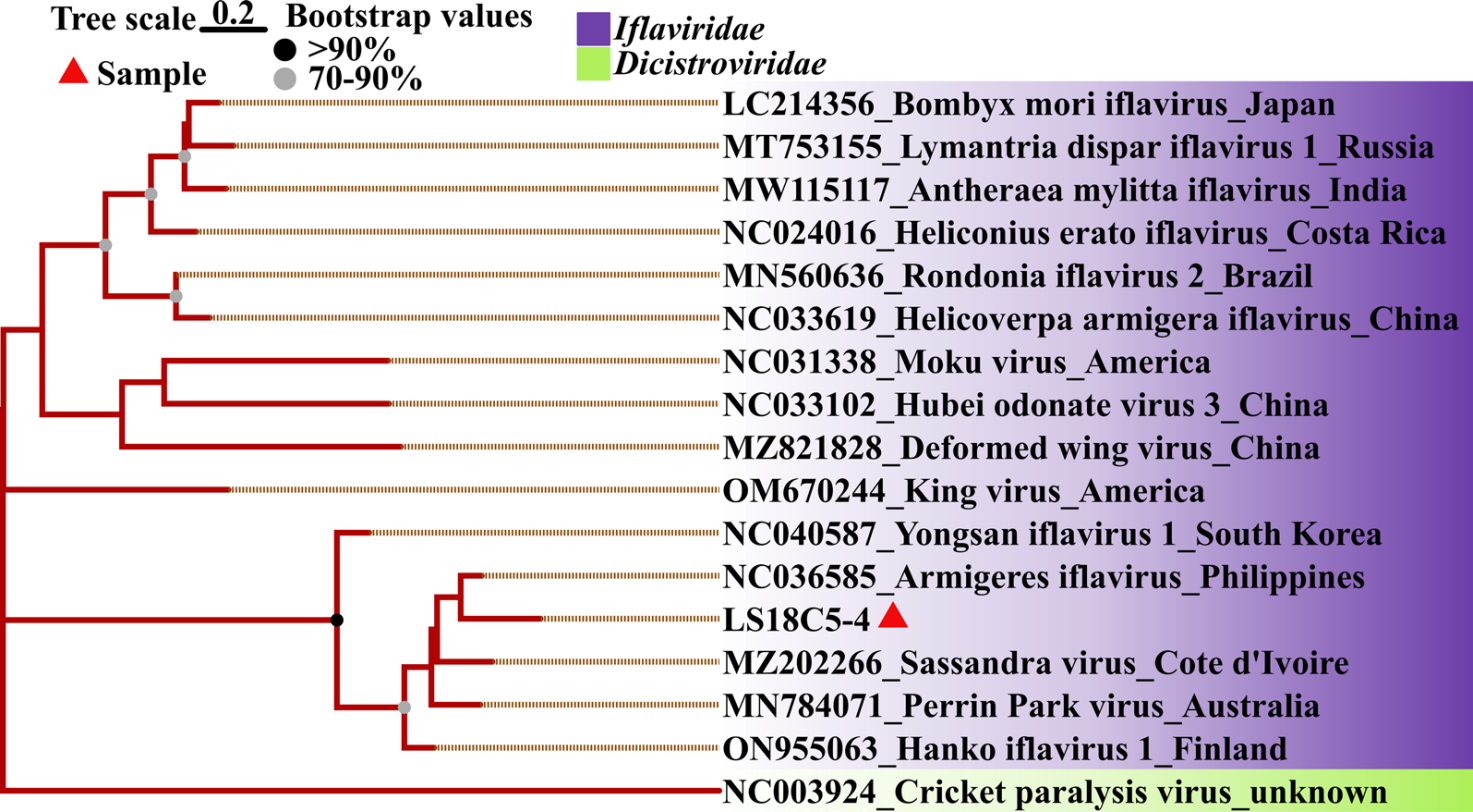
Maximum-likelihood phylogenetic tree based on partial amino acid sequences of the RNA-dependent RNA polymerase (RdRp) of Armigeres iflavirus (ArIFV) and related viruses in the family *Iflaviridae*. The red triangle indicates the newly isolated ArIFV-related strain identified in this study. The best-fit maximum-likelihood evolutionary model was the Whelan and Goldman model with gamma-distributed rate heterogeneity (WAG + G). Bootstrap values were calculated from 1000 replicates

For ArIFV identification, strain LS18C5-4 was isolated from a pool of *C. arakawai* and amplified using primers targeting the Simbu group of bunyaviruses. BLASTx analysis revealed 76.0% amino acid identity with Armigeres iflavirus (ArIFV; GenBank: NC036585), which was isolated in the Philippines in 2010. A phylogenetic tree was constructed using partial amino acid sequences of the RdRp gene from strain LS18C5-4, 15 reference strains from the family *Iflaviridae*, and 1 strain from the family *Dicistroviridae*. Phylogenetic analysis showed that LS18C5-4 clustered within *Iflaviridae*, specifically in the same branch as ArIFV, indicating that it is a member of this family and is most closely related to ArIFV (Fig. 11).

## Discussion

The western border region of Yunnan Province, where many arboviruses reported in China have been detected, adjoins Myanmar, where multiple arboviruses are endemic. Characterized by a hot, humid climate with abundant rainfall throughout the year, the region has an average annual temperature ranging from 16 °C to 28 °C, providing favorable ecological conditions for the reproduction and transmission of arbovirus vectors. In this study, eight mosquito species belonging to four genera—*Culex*, *Anopheles*, *Armigeres*, and *Aedes*—were collected from Mangshi, Yingjiang, Lushui, and Tengchong. *Cx. tritaeniorhynchus* was identified as the dominant species in Mangshi and Yingjiang, whereas *An. sinensis* predominated in Lushui and Tengchong. Both species are major vectors of several important pathogens, including JEV [20], Getah virus (GETV) [21], and *Plasmodium* [22]. They exhibit nocturnal host-seeking behavior, frequently feed on humans and livestock, and are commonly found in peridomestic environments and human dwellings [23]. A total of 189 viral strains were isolated in this study, with the majority originating from Mangshi (163 strains), followed by Lushui (17 strains), Tengchong (6 strains), and Yingjiang (2 strains). These findings indicate that the border region harbors a diverse range of vector species and exhibits a high prevalence of arbovirus carriage.

Yunnan orbivirus (YUOV), Tibet orbivirus (TIBOV), and Banna virus (BAV) are members of the family *Sedoreoviridae*. Among them, YUOV and TIBOV belong to the genus *Orbivirus* and possess genomes composed of ten segments of double-stranded RNA. YUOV was first isolated from *Cx. tritaeniorhynchus* in Yunnan Province in 2005 and has been shown to induce neurological symptoms in animals [23]. The detection of immunoglobulin (Ig)G and IgM antibodies against YUOV in human serum samples collected from Xishuangbanna Prefecture, Yunnan, in 2011 suggests its potential for human infection [14]. In the present study, 33 strains of YUOV were isolated in Mangshi, with 20 originating from *Cx. tritaeniorhynchus* and 13 from *C. tainanus*. These findings suggest a potential role for *Culicoides* in the transmission of YUOV, while *Cx. tritaeniorhynchus* may also be involved in the transmission cycle of the virus in this region. Further experimental infection studies are needed to confirm vector competence.

TIBOV, recognized as an orbivirus, was first identified in Tibet, China, in 2009 [24]. Infection with TIBOV can cause fever and anorexia in domestic animals and can lead to death in suckling mice within 72 h [25]. A total of 83 TIBOV strains were isolated in this study, including 30 from *Cx. tritaeniorhynchus*, 2 from *Cx. pseudovishnui*, 1 from *An. sinensis*, and 50 from *Culicoides* spp. TIBOV not only represented the highest number of isolates in this survey, but was also detected in all four sampling regions, indicating its wide distribution across Yunnan Province and its ability to circulate among multiple host and vector species.

Banna virus (BAV) is a virus in the genus *Seadornavirus* and an important pathogen associated with viral encephalitis. It was first isolated in 1987 from the serum of patients with fever and the cerebrospinal fluid of patients with encephalitis in Xishuangbanna [17]. In this study, six BAV strains were obtained from Lushui, including two A1-type strains isolated from *Cx. tritaeniorhynchus* and four A2-type strains from *C. arakawai*. This suggests that different vector species within the same locality may carry distinct BAV genotypes. Given that *Cx. tritaeniorhynchus* is the dominant mosquito species in the study areas and was found to harbor BAV, it may play a role in the natural transmission cycle of the virus.

Akabane virus (AKAV) belongs to the Simbu serogroup within the genus *Orthobunyavirus* of the family *Peribunyaviridae*. It is a segmented, single-stranded, negative-sense RNA virus classified into four genotypes (I–IV), with genotype I further divided into subtypes Ia and Ib [26]. AKAV has been reported in Australia, Asia, and the Middle East [27]. Here, we identified one AKAV strain from *C. arakawai* in Lushui, which clustered within genotype Ia along with six other Chinese strains, suggesting that genotype Ia may be the predominant genotype circulating in China. AKAV is known to infect a variety of animals and can cause abortion and stillbirth in pregnant animals [28]. Although the virus has occasionally been isolated from mosquitoes, *Culicoides* are considered its primary vectors [29]. The widespread distribution of *Culicoides* in Lushui may facilitate the continued transmission of AKAV in the region.

A total of 64 insect-specific virus (ISV) strains were identified, representing 4 viral groups. NDiV, classified within the genus *Alphamesonivirus*, family *Mesoniviridae*, and order *Nidovirales*, is an unsegmented, single-stranded, positive-sense RNA virus and represents one of the largest groups of nonsegmented RNA viruses infecting insects [30]. In this study, four NDiV strains were isolated from Lushui, including one from *An. sinensis* and three from *Culicoides* spp., confirming that *Culicoides* may also serve as important vectors for NDiV. MAV, a single-stranded, positive-sense RNA virus, belongs to the negevirus group [31]. We isolated 53 MAV strains from mosquitoes and *Culicoides* in Mangshi and Lushui, representing the first detection of MAV in *Culicoides* and its first report in both Mangshi and Lushui. CppDNV is a small, non-enveloped, single-stranded DNA virus in the family *Parvoviridae* [32]. In this study, two CppDNV strains closely related to CxDNV—but more distantly related to HeDNV and AalDNV—were isolated from *Cx. tritaeniorhynchus* and *C. tainanus* in Mangshi. CxFV is a single-stranded, positive-sense RNA virus in the family *Flaviviridae* [33]. Two CxFV strains closely related to YCxFV previously identified in Yunnan were isolated from *Cx. tritaeniorhynchus* in Mangshi. Negeviruses have been demonstrated to suppress arbovirus replication in mosquitoes both in vivo and in vitro [34]. Consequently, coinfection of insect-specific viruses (ISVs) and arboviruses has emerged as a research hotspot, offering a novel strategy for arbovirus control. Furthermore, biological control approaches based on ISVs may eventually reduce reliance on chemical insecticides and contribute substantially to the prevention of arboviral diseases.

The family *Tymoviridae* comprises single-stranded, positive-sense RNA viruses transmitted by phytophagous insects during feeding. These viruses primarily infect dicotyledonous and graminaceous plants—such as turnip, cabbage, maize, and grape—and can cause considerable agricultural losses [35]. The MS18M129-29 strain identified in this study clustered within the genus *Maculavirus* and showed amino acid similarities of 61.64%, 49.32%, 52.86%, and 51.25% with CuTLV-J9, BtLV-UW1, EkBV-2007 (Mex), and MuTV-BeAr855909, respectively, suggesting that MS18M129-29 may represent a new member of the genus *Maculavirus*. The family *Iflaviridae* comprises small, non-enveloped, single-stranded, positive-sense RNA viruses [36]. The genus *Iflavirus* is the only taxon within this family; these viruses infect arthropods and can cause behavioral changes, developmental abnormalities, and premature death in economically important insects [37]. Here, the LS18C5-4 strain was isolated from *C. arakawai* in Lushui and clustered within *Iflaviridae*. It shared amino acid identities of 76.03%, 71.07%, and 73.55% with ArIFV-10P38-310, HaIFV1-FIN/L-2018/24, and PPKV-205642, respectively, suggesting that LS18C5-4 may be a novel member of *Iflaviridae*.

This study led to the isolation of multiple viruses from the western border region of Yunnan. These included human pathogens such as BAV, which is associated with febrile illness and encephalitis, as well as veterinary pathogens such as YUOV, TIBOV, and AKAV. Although YUOV-specific antibodies have been detected in humans, the potential health risks require further investigation. Additionally, two novel viruses belonging to the families *Tymoviridae* and *Iflaviridae*—which may negatively affect crops and beneficial insects—were identified. Four insect-specific viruses (ISVs), namely NDiV, MAV, CppDNV, and YCxFV, were also detected. In recent years, the influence of ISVs on pathogenic arboviruses in mosquitoes has attracted considerable scientific interest, providing a promising direction for the biological control of arboviral diseases.

This study has several limitations that should be considered when interpreting the findings. First, the sampling was conducted during a single 4-day period in July 2018, which may not capture the seasonal dynamics or full diversity of arboviruses circulating in the region. As such, the results represent a temporal snapshot rather than a comprehensive assessment of arbovirus prevalence, and broader conclusions regarding distribution, diversity, or vector importance require validation through longitudinal surveys spanning multiple seasons and years. Second, virus identification relied primarily on RT-PCR and sequencing, without additional purification or phenotypic characterization of the isolates. Consequently, confirmation of infectious virus particles necessitates further studies involving plaque purification and in vitro characterization. Moreover, the detection of viral RNA does not unequivocally indicate active replication within the vectors; experimental infection studies are needed to definitively establish vector competence.

## Conclusions

This study demonstrates that the western border region of Yunnan harbors a diverse and abundant population of mosquitoes and biting midges, and that these insects carry a wide spectrum of viruses during the sampling period. These include both previously recognized arboviruses and novel viral strains that warrant further characterization. Our investigation of midge-borne viruses in this area contributes to the understanding of local vector-borne infectious diseases. The findings provide valuable baseline information that can inform vector surveillance and control efforts against insect-borne infectious diseases in China. However, given the limited sampling duration, further longitudinal studies are needed to fully elucidate the seasonal dynamics and long-term patterns of arbovirus transmission in this region.

## Supplementary Information

Supplementary Material 1.

## Data Availability

All the data generated or analyzed during this study are included in this published article.
