# Supplementary material for: Mosquito- and biting-midge-borne arboviruses in Western Yunnan’s border region, China
Source: Parasit Vectors. 2026 May 27;19:297. doi: 10.1186/s13071-026-07400-6 (PMC13393271; doi:10.1186/s13071-026-07400-6)
Supplement: Supplementary file 1 — Supplementary Material 1. [file 13071_2026_7400_MOESM1_ESM.docx]

Table S1. Primer information

| **Virus** | **Primer** | **Primer sequence (5'- 3')** | **Product length (bp)** | **Annealing Temperature (℃)** | **Extension Time (s)** |
| --- | --- | --- | --- | --- | --- |
|  |  |  |  |  |  |
| Yunnan orbivirus | YUOV S10F | AGCATTCGGTACGCAGTATCTCG | 831 | 53 | 45 |
|  | YUOV S10R | GCCGAGCCGATCATGTCACGTGT |  |  |  |
| Manglie virus | NGV1094F | GTCCCACCCTTTCAGAGT | 1094 | 50 | 75 |
|  | NGV2287R | AAGTTGTGAGTAGGTTTGAGA |  |  |  |
| Culex-originated Tymoviridae-like virus | KHV S10F | TATTCTAACCAACGCAGTTACCTCA | 500 | 60 | 40 |
|  | KHV S10R | TCCAATGCTGGCGCTGAA |  |  |  |
| Tibet orbivirus | TIBOV S7F | TTTAGCGGCAGCAAACATCA | 487 | 52 | 30 |
|  | TIBOV S7R | TCATTCCATTCCAGGCAACC |  |  |  |
| Banna virus and  Nam Dinh virus | 12-854-S | AAATTGATAGYGYTTGCGTAAGAG | 845 | 57 | 60 |
|  | 12-B2-R | GTTCTAAATTGGATACGGCGTGC |  |  |  |
| Akabane virus and  Armigeres iflavirus | Simbu 3F | TCTTCTTCCTAAYCAGAAGAA | 396 | 54 | 40[20] |
|  | Simbu 2R | ACCTTCCTCATGAAGTTGACA |  |  |  |
|  | Simbu 11F | GGTTAATAACCATTTTCCCCA |  |  |  |
| Yunnan Culex flavivirus | Ful | TACCACATGATGGGAAAGAGAGAGAA | 310 | 55 | 30[21] |
|  | cFD2 | GTGTCCCAGCCGGCGGTGTCATCAGC |  |  |  |
| Densovirus | DNVS1F | GGTGATTCTGGTTCTGACTCTTG | 1035 | 53 | 75[22] |
|  | DNVS1R | GARRGVCARAGYGAGWDATG |  |  |  |
